# A case of light chain deposition disease involving the kidney with a normal serum free kappa/lambda light chain ratio

**DOI:** 10.1080/0886022X.2021.2021943

**Published:** 2022-02-14

**Authors:** Suojian Zhang, Haifeng Ni, Qin Xu, Xiaoqin Cai, Haitao Li, Zhiqiang Wei, Juan Cao

**Affiliations:** a Department of Nephrology, Taixing People’s Hospital, Taizhou, China; b Department of Nephrology, Zhongda Hospital Southeast University, Nanjing, China

Dear Editor,

Light chain deposition disease (LCDD) is a monoclonal gammopathy-related disease, which can cause damage to many organs, including the liver, heart, lung, and kidney. The kidney is one of the most commonly affected organs [1–3]. Patients with LCDD involving the kidney may present with proteinuria, hematuria, nephrotic syndrome, and renal impairment [4]. LCDD can be secondary to multiple myeloma and plasma cell disease, or it may not be associated with any other disease. In 2012, the International Kidney and Monoclonal Gammopathy Research Group named the condition of monoclonal gammopathy without plasma cell disease or B lymphocyte proliferative disease, but with renal damage, as monoclonal gammopathy of renal significance (MGRS) [5]. LCDD is a rare disease and previously reported cases have had significantly increased or decreased serum free kappa/lambda ratios [1,6,7]. Here, we report a case of LCDD involving the kidney with a normal serum-free kappa/lambda ratio.

## Case presentation

**Figure 1.**
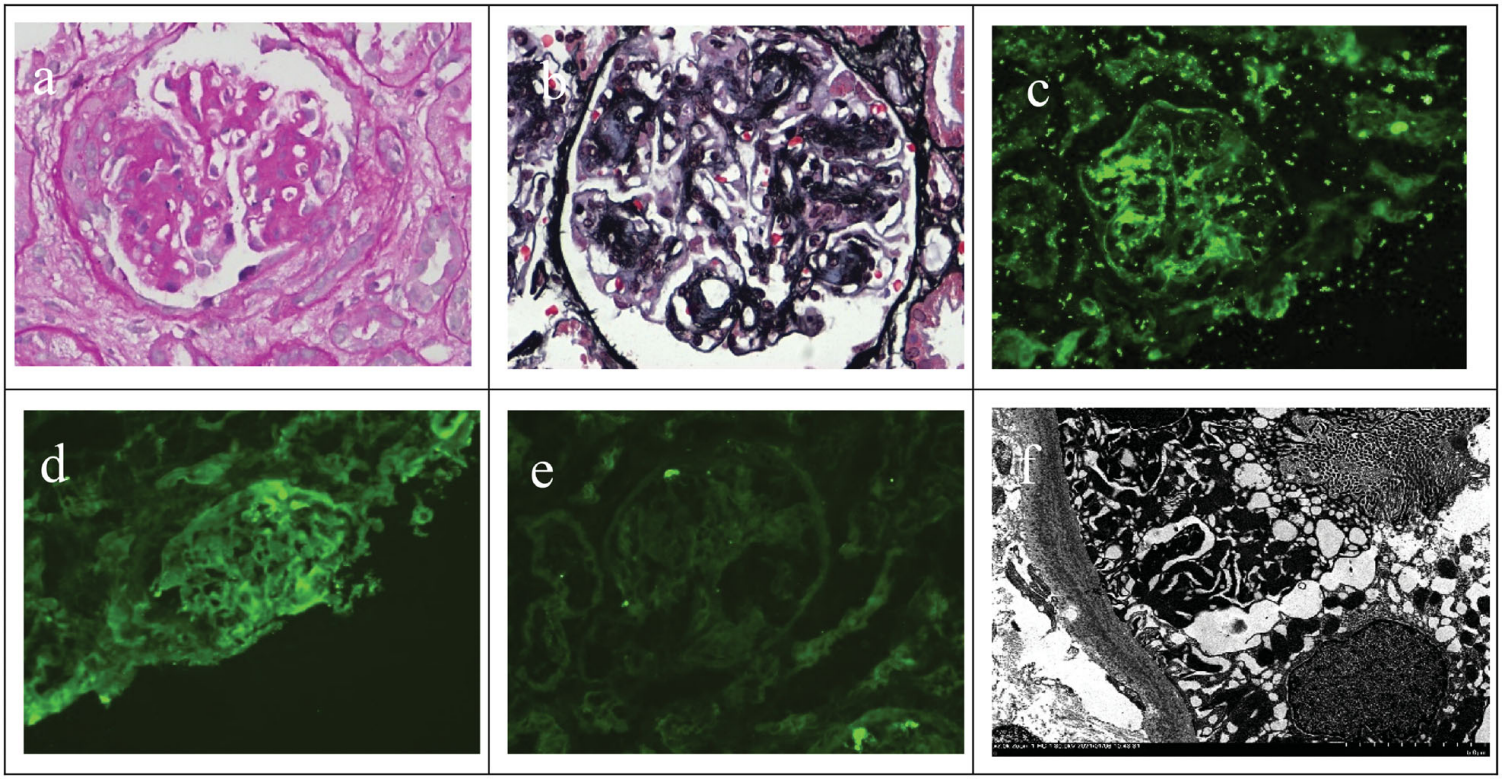
(a,b) Light microscopy; (c) immunofluorescence C3+++; (d) immunofluorescence kappa+; (e) immunofluorescence lambda-; and (f) electron microscopy.

A 53-year-old female patient was admitted to our department on 24 December 2020 because of ‘edema for more than 1 month’. One month before admission, the patient presented with edema of the face and lower limbs accompanied by foamy urine. She had a history of hypertension for more than 2 years with blood pressure as high as 185/100 mmHg. Her laboratory test results included: hemoglobin 112 g/L, urine protein 2+, urine red blood cell 242/uL, albumin 33.6 g/L, creatinine 67 μmol/L, urea 7.07 mmol/L, potassium 4.04 mmol/L, sodium 143.0 mmol/L, calcium 2.1 mmol/L, and phosphorus 1.4 mmol/L. Serum-free kappa light chain 24.29 mg/L (3.3–19.4), serum-free lambda light chain 33.76 mg/L (5.71–26.3), serum-free kappa/lambda light chain ratio 0.7195 (0.26–1.65), 24 h urine protein 2.4 g. Blood glucose, glycated hemoglobin, anti-ENA spectrum, tumor markers, ANCA, C3 and C4 levels were all normal. HBV, HCV, syphilis, and HIV tests were negative. B-mode ultrasound showed that both her kidneys were normal in size, with enhanced echogenicity in the parenchymal area. After admission to our department, chronic glomerulonephritis was considered as a potential diagnosis and renal biopsy was performed on 29 December 2020. Light microscopy showed moderate diffuse hyperplasia of endothelial cells, mesangial cells and stroma, severe hyperplasia of focal segments, nodular changes and subcutaneous insertion, and obvious capillary lobulation. Multifocal renal tubular atrophy was also present (Figure 1(a–b)). Immunofluorescence: C_3_+++; IgG, IgA, IgM, C1q−; Kappa+, lambda−. Deposition site: mesangial region of the glomeruli. Deposition mode: granular (Figure 1(c–e)). Electron microscopy showed deposition of silt-like electron densities in the medial basement membrane of glomerular capillaries, the mesangial area and the lateral basement membrane of renal tubules (Figure 1(f)). Based on the clinical, immunofluorescence, light and electron microscopy results, the patient was diagnosed with LCDD involving the kidney.

Further examination of urine Bence-Jones protein was negative and no M protein was found in urine protein electrophoresis. Serum protein electrophoresis found a suspected M protein band but serum immunofixation electrophoresis found no abnormalities. Bone marrow biopsy showed that the proportion of plasma cells was 3.5% and the proportion of granulocytes and red cells was normal. Bone marrow Congo red staining was negative. The patient's bone marrow plasma cells were analyzed by flow cytometry and monoclonal plasma cells were found.

**Figure 2.**
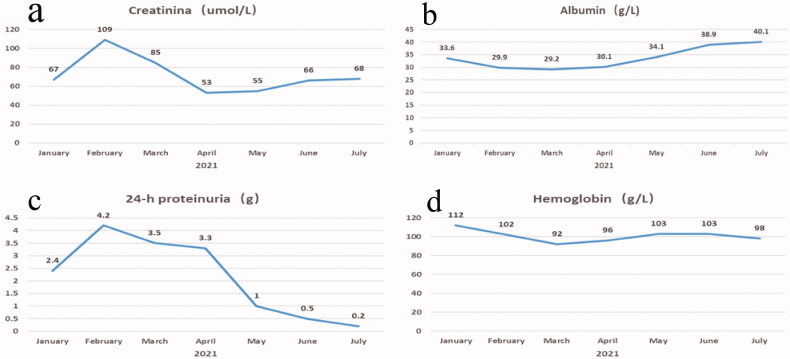
(a) Creatinine; (b) albumin; (c) 24 h urine protein; and (d) hemoglobin.

The treatment prescribed was bortezomib 1.3 mg/m^2^ combined with dexamethasone 20 mg, once a week, every four times as a course of treatment. Chemotherapy was started on February 9th and five courses of treatment have been completed so far. After treatment, the patient’s edema disappeared, her urinary protein decreased, and her renal function returned to normal. Her creatinine, serum albumin, 24-h urine protein, and hemoglobin levels during follow-up are shown in Figure 2(a–d).

## Discussion

LCDD involving the kidney is a relatively rare disease, which was first described by Randall in 1976 [8]. LCDD involving the kidney tends to occur in people aged 50–60 and its clinical manifestations are hematuria, proteinuria, renal insufficiency, and hypertension [9]. In this case, the patient presented with hematuria, proteinuria, and hypertension. Her renal function was normal on presentation, but her serum creatinine increased during follow-up, and recovered after treatment. Multiple myeloma is one of the most common causes of LCDD [10]. However, some LCDD patients do not have myeloma or any other malignant hematological disease. In this case, the patient underwent bone marrow biopsy to screen for malignant hematological diseases.

Renal biopsy is important for the diagnosis of LCDD. The main histopathological manifestations of LCDD are mesangial nodular changes. In this case, the patient’s renal pathology showed nodular changes. Furthermore, immunofluorescence showed that kappa light chains were deposited in the glomerular mesangium. Electron microscopy found depositions of silt-like electron densities in the medial basement membrane of glomerular capillaries, the mesangial area and the outer side of the basement membrane of the renal tubules, supporting a diagnosis of LCDD. Although silt-like electron densities were observed in the lateral basement membrane of renal tubules, immunofluorescence did not find kappa light chain deposition in this location, which may be due to the focal distribution of the lesions.

Abnormal serum-free kappa/lambda ratios have been previously reported in LCDD patients [1,6,7]. In 2012, Nasr et al. reported 64 cases of monoclonal immunoglobulin deposition disease at the Mayo Clinic, including 51 cases of LCDD, and all patients who underwent serum free light chain detection had abnormal serum free kappa/lambda ratios [9]. In this case, the patient’s serum-free kappa/lambda ratio was normal on presentation and no obvious abnormality in the bone marrow was found on biopsy. However, serum protein electrophoresis found a suspected M protein band, and the diagnosis of LCDD was confirmed by renal biopsy. This emphasizes the importance of renal biopsy in the diagnosis of potential LCDD patients. In this case, the diagnosis of LCDD may have been missed if renal biopsy had not been performed.

There is currently no standardized treatment plan for LCDD. Ziogas used a bortezomib-based treatment regimen to treat LCDD patients and 61% achieved some level of remission [11]. Batalini treated LCDD patients with high-dose melphalan and stem cell transplantation, which showed a good effect [12]. In recent years, rituximab has also been reported to have a good effect on LCDD [13,14]. In this case, bortezomib combined with dexamethasone achieved good results, but further follow-up is required to assess the patient’s long-term prognosis.

## Data Availability

All data are included in the manuscript.
